# Role of Fat and Bone Biomarkers in the Relationship Between Ethnicity and Bone Mineral Density in Older Men

**DOI:** 10.1007/s00223-017-0342-8

**Published:** 2017-10-20

**Authors:** Grace M. F. Chan, Tessa Riandini, Sheryl Hui Xian Ng, Su Yen Goh, Chuen Seng Tan, E. Shyong Tai, Gustavo Duque, Alvin Choon-Meng Ng, Kavita Venkataraman

**Affiliations:** 1 Yong Loo Lin School of Medicine, National University of Singapore, 1E Kent Ridge Road, Level 11, NUHS Tower Block, Singapore, 119228 Singapore; 2 Saw Swee Hock School of Public Health, National University of Singapore, #10-03 J, Level 10, Tahir Foundation Building (MD1), 12 Science Drive 2, Singapore, 117549 Singapore; 3 Department of Endocrinology, Singapore General Hospital, Academia, Level 3, Outram Road, Singapore, 169608 Singapore; 4 Australian Institute for Musculoskeletal Science (AIMSS), University of Melbourne and Western Health, St. Albans, VIC Australia; 5 The Endocrine Clinic, Mount Elizabeth Medical Centre, 3 Mount Elizabeth, #15-04, Singapore, 228510 Singapore; 6 Singapore General Hospital, Singapore, Singapore

**Keywords:** Bone mineral density, Adipokines, Bone biomarkers, Adiposity, Osteoporosis

## Abstract

Osteoporosis is an important health issue for older adults, and has been relatively understudied in older men. This study aimed to examine ethnic differences in bone mineral density (BMD), and elucidate the role of bone turnover markers (BTMs), fat and fat biomarkers on these ethnic differences. BMD at the lumbar spine and femoral neck, marrow fat at femoral neck, visceral adipose tissue (VAT) and subcutaneous adipose tissue, bone and fat biomarkers were evaluated in 120 healthy men aged ≥ 60 years. Indians had higher BMD values compared to Chinese at the lumbar spine (*β* = 20.336, SE = 4.749, *p* < 0.001) and the femoral neck (e^*β*^ = 1.105, SE = 0.032, *p* < 0.001), after adjusting for BTMs, fat composition and lifestyle choices. Marrow fat, VAT and adiponectin were independent predictors of BMD. However, these factors did not explain the lower BMD observed in older Chinese men. Our findings suggest that older Chinese men are at significant risk of osteoporotic fractures due to lower BMD. Fat appears to be a key factor associated with lower BMD, and warrants further longitudinal studies to elucidate the complex interactions between adipose tissue and bone strength.

**Electronic supplementary material:**

The online version of this article (doi:10.1007/s00223-017-0342-8) contains supplementary material, which is available to authorized users.

## Introduction

Osteoporosis is a growing public health challenge worldwide, accounting for approximately 50% of all hip fractures [1] and 5.8 million disability-adjusted life years annually [2]. Low bone mineral density (BMD) accounted for an estimated 3.7 million years lived with disability in 2015, an increase of 53% from 1990 [3]. Individuals with osteoporosis are at increased risk of fracture after relatively minor falls, and the elderly are particularly vulnerable, as bone loss progresses with age [4]. With its rapidly ageing population, Asia-Pacific will bear the brunt of this problem, and 50% all hip fractures are expected to occur in this region by 2050 [5]. Singapore, specifically, has the highest reported incidence of hip fractures in Asia [6].

There are substantial geographic and ethnic variations in fracture rates around the world. For both men and women, the highest age-adjusted hip fracture rates have been reported in North Europe and America, and lowest in Africa [7]. Among ethnic groups, Caucasians have the highest rates of hip fracture compared to other ethnic groups [7]. These differences can, at least partly, be attributed to the differences in BMD across ethnic groups [8]. Multiple studies within the US have shown that African Americans have the highest BMD while Caucasians have the lowest [9, 10]. We, and others, have previously demonstrated substantial ethnic variation in hip fracture incidence and BMD in Singapore, with Chinese having the highest hip fractures rates, in both men and women [11], as well as the lowest BMD [12]. Beyond BMD, the ethnic difference in fracture risk may also be due to the differences in bone size and bone geometry, such as femoral neck size, trabecular and cortical thickness and bone volume [13, 14].

Other factors may also be involved as drivers of ethnic differences in fracture risk and BMD. Fat distribution and bone marrow adipose tissue may play a role in bone turnover [15, 16]. In Singapore, fat distribution differs among ethnic groups [17], and studies have demonstrated that Chinese accumulate higher levels of visceral adipose tissue (VAT) with greater adiposity than Indians [18]. Several mechanisms have been postulated about the fat–bone relationship: the mechanical loading of fat mass on bone, the paracrine effect of hormones from the pancreatic cell (such as insulin, amylin, preptin) acting on bone, the endocrine effect of adipocyte-secreted hormones (such as adiponectin, leptin) and the release of inflammatory markers (interleukin-6) [19]. Indeed, leptin has been reported as being positively correlated, and adiponectin negatively correlated with BMD [20]. In addition, measuring bone turnover markers (BTMs) and inflammatory factors might also be helpful in the understanding of bone metabolism in different ethnic groups. BTMs can serve as surrogates for monitoring osteoblastic and osteoclastic activity [21]. Inflammation has been causally implicated in pathways to bone loss [22] and is associated with increased risk of incident fractures [23].

Hence, understanding ethnic influences on the pathophysiology of osteoporosis is important to decrease its impact in an ageing population. This could guide screening tools in at-risk ethnic groups, as well as help design new interventions targeted to prevent and treat osteoporosis. Therefore, the aims of this present study were to examine the ethnic differences in BMD between older Chinese and Indian men, and elucidate the role of BTMs, fat and fat biomarkers as potential determinants of these ethnic differences.

## Methods

This was a cross-sectional study of 120 apparently healthy men (60 Chinese and 60 Indian) aged 60 years and above. Ethical approval was obtained from the Singapore General Hospital Institutional Review Board before commencement of the study, and all study procedures were conducted in accordance with relevant guidelines and regulations. Written informed consent was obtained from all participants prior to their participation.

Participants were consecutively recruited from the attendees of community-based health fairs, based on eligibility and willingness to participate. A detailed medical history was obtained from all participants on history of chronic diseases, including diabetes, hypertension and ischaemic heart disease, and lifestyle, including smoking, alcohol use, physical activity and sun exposure. Subjects with diabetes mellitus, Cushing’s syndrome, on antiviral/anti-obesity/steroids/anti-osteoporosis drugs, previous abdominal surgery, previous cancer, any investigational drugs for the past 3 months or excessive weight loss (> 5% body weight) over the last 3 months were excluded from the study.

Height (to the nearest millimetre) was recorded in all subjects without shoes, and weight (in kilograms) was measured with subjects in light clothing using electronic weighing scales (SECA model 220) to compute BMI (weight in kilograms divided by the square of the height in metres).

### Biochemical Analysis

Fasting blood specimens were obtained from all respondents after an overnight fast of 10 h for all analytes. Plasma glucose and lipid measurements (total cholesterol, triglyceride, HDL cholesterol), serum albumin, C-reactive protein and inorganic phosphate were analysed on the same day as collection (Beckman Coulter UniCel DxC System, Beckman Coulter, Inc., USA). LDL cholesterol was calculated by Friedewald’s equation. For all other analytes, blood samples were stored as serum or plasma at – 80 °C prior to assay. 25-Hydroxy vitamin D was measured by radioimmunoassay (DiaSorin, Italy). The adipokines leptin, resistin and adiponectin were measured using commercially available kits (Linco Research, Inc., USA). Serum insulin and intact PTH were measured by immunoassay (Beckman Coulter UniCel DxI System, Beckman Coulter, Inc., California, USA). Plasma osteocalcin and serum procollagen type 1 n-terminal propeptide (P1NP) were measured by immunoassay (Roche Cobas 6000 analyzer, Roche Diagnostics, USA). These analytes were measured at the laboratory at Singapore General Hospital, which is Joint Commission International and College of American Pathologists Laboratory Accreditation Programme accredited. Interferon γ, interleukin-1α (IL1α), interleukin-1β, interleukin-6, macrophage inflammatory protein-1α, receptor activator of nuclear factor κ-B ligand, tumor necrosis factor α, insulin-like growth factor-1, carboxy-terminal collagen crosslinks, osteopontin and osteoprotegerin were measured using flow cytometry (Luminex 100/200 System for multiplex assays, tests run by i-DNA Biotechnology, Singapore). The intra-assay and inter-assay coefficients of variation for the analytes measured by Luminex ranged from 6.2 to 21.8 and 6.9 to 19.8, respectively.

### Image Acquisition and Analysis

Scanning for measurement of BMD was performed on a 64-slice multi-detector dual source CT scanner (Somatom Definition, Siemens AG, Erlangen, Germany). Axial CT scans were performed with the subjects supine, from the dome of the diaphragm down to the bottom of the pelvis, using a 35 × 35 cm field of view. All scans were non-contrast enhanced and used routine scan parameters: kVp 120, effective mAs 210, slice collimation 0.6 mm, slice width 5.0 mm, pitch factor 1.4 and increment 5.0 mm. Calculation of bone mass and bone volumes at the lumbar spine (at L2) and left femoral neck was done by correlation with scans of phantoms pre-calibrated against the CT scanner using QCT Pro (MindwaysCT), and volumetric BMD at lumbar spine (LS-BMD) and femoral neck (FN-BMD) calculated. All image analysis was performed under the guidance of the radiologist.

The same scan images were used to determine VAT and subcutaneous adipose tissue (SAT) at L2/3 vertebral level, and marrow fat at the femoral neck. VAT was assessed according to the method proposed by Shen et al. [24], based on the cross-sectional area of the adipose tissue within the inner edge of the abdominal wall at L2/3 vertebral level, whereas SAT was assessed based on the cross-sectional area of the adipose tissue within the outer edge of the abdominal muscular wall. Adipose tissue was indicated by lower density areas on the CT images with attenuation values of − 195 to − 45 Hounsfield units [25]. Manual editing of the images was then done to exclude faecal matter and bowel gas from the calculations. Marrow fat was analysed using axial slices of the left femoral neck (sliceOmatic, Tomovision™, Montreal, Canada), with the adequate threshold of signal intensity calculated from an average of 20 quantifications.

### Statistical Methods

CT- measured volumetric LS-BMD and FN-BMD were the outcomes of interest. Normality of BMD outcomes was tested using a Shapiro–Wilk test. FN-BMD was log-transformed to fulfil the normality assumption.

Other potential independent variables of interests were bone biomarkers, fat and fat biomarkers. Biomarkers were presented as quintiles and fat variables were standardised. All other variables were considered as potential confounders. Bivariate associations between the BMD outcomes and each variable of interest, and potential confounders were tested, and the variables exhibiting association of *p* < 0.2 were shortlisted for model-building (Supplementary Table 1).

Linear models for BMD measures were set up with ethnicity, each variable of interest and confounders as covariates. The effect of ethnicity in each model was compared to that of the bivariate association and across the different confounders. A final model was constructed for each BMD outcome by adjusting for demographic confounders, pooling all variables of interest and running a stepwise variable selection procedure. Similarly, the effect of ethnicity on BMD was of interest and compared across the models. *β* Coefficients were reported for models of LS-BMD, with coefficients representing the magnitude of change in LS-BMD in mg/cm^3^ for unit change in specified independent variable(s). Exponentiated *β* coefficients were reported for FN-BMD models as a measure of association on the original scale of measurement, with coefficients representing the value by which FN-BMD is multiplied for unit change in specified independent variable(s).

## Results

### Study Population

The 120 men involved in our study (60 Chinese, 60 Indian) were from 60 to 89 years of age, with mean BMI of 25.21 (± 4.45) kg/m^2^. Differences in age and BMI were significant between the two ethnic groups (*p* value of 0.020 and < 0.001, respectively), with Indian participants being slightly younger and having greater BMI. There were no differences between the two groups in terms of family history of fragility fracture or previous history of fracture. Table 1 summarises the relevant characteristics of the study population.

**Table 1:** Characteristics of participants in the study

Variables	Total	Chinese (*n* = 60)	Indian (*n* = 60)	*p* values*
	Mean (SD)			
Age (years)	66.24 (5.97)	66.8 (4.71)	65.68 (7.00)	0.020
BMI (kg/m^2^)	25.21 (4.45)	24.14 (4.32)	26.28 (4.34)	< 0.001
Height (cm)	164.68 (6.72)	164.18 (6.44)	165.18 (7.01)	0.293
LS-BMD^a^ (mg/cm^3^)	119.48 (27.63)	109.14 (27.34)	129.48 (24.20)	< 0.001
FN-BMD^b^ (mg/cm^3^)	0.69 (0.12)	0.65 (0.12)	0.72 (0.10)	< 0.001

	*n* (%)			
Smoking status				
Never smoked	90 (75)	42 (70.00)	48 (80.00)	0.084
Ex-smoker	25 (20.83)	17 (28.33)	8 (13.33)	
Smoker	5 (4.17)	1 (1.67)	4 (6.67)	
Sun exposure score^c^				
Low	84 (73.04)	49 (81.67)	35 (58.33)	0.008
Moderate	21 (18.26)	5 (8.33)	16 (26.67)	
High	10 (8.7)	3 (5.00)	7 (11.67)	
Cerebrovascular disease	5 (4.17)	0 (0)	5 (8.33)	0.057
Hypertension	39 (32.5)	13 (21.67)	26 (43.33)	0.019
Diabetes mellitus^b^	20 (17.09)	1 (1.67)	19 (33.33)	< 0.001
Dyslipidemia^b^	34 (29.06)	10 (16.95)	24 (41.38)	0.004
History of fracture	24 (20)	12 (20)	12 (20)	1.000

	Median (IQR)			
Total activity level	77.44 (35.03, 155.79)	86.84 (50.18, 165.54)	68.69 (28.94, 134.54)	0.038
Physical activity level: light	49.32 (24.21, 100.45)	50.73 (25.83, 101.58)	47.79 (21.51, 100.45)	0.492
Physical activity level: moderate	12.75 (1.61, 47.92)	20.62 (7, 67.53)	3.93 (0.23, 27.18)	< 0.001
Physical activity level: vigorous	0 (0, 5.63)	0 (0, 12.38)	0 (0, 2.13)	0.287

Biomarkers	Median (IQR)			
Vitamin D (µg/L)	21.65 (18.9, 24.75)	24.15 (22.3, 30.15)	19.9 (15.9, 21.2)	< 0.001
Parathyroid hormone (pmol/L)	4.1 (3.4, 5.25)	3.8 (3.25, 4.35)	3.8 (3.4, 4.6)	0.068
Phosphate^d^ (mmol/L)	0.98 (0.92, 1.08)	0.95 (0.9, 1.06)	1.02 (0.87, 1.18)	0.248
Albumin^d^ (g/L)	38 (36, 40)	39 (37, 40)	37 (36, 40)	0.123
Interferon γ (IU/mL)	17.59 (10.84, 26.99)	16.44 (10.14, 26.21)	27.49 (17.19, 32.66)	0.038
Interleukin-1 α (pg/mL)	6.02 (4.91, 7.4)	5.17 (3.9, 6.71)	7.37 (6.13, 9.39)	< 0.001
Interleukin-1 β (pg/mL)	1.39 (1.16, 2.05)	1.98 (1.28, 2.08)	2.01 (1.22, 2.31)	0.235
Interleukin-6 (IU/mL)	4.6 (3.53, 5.5)	4.73 (3.71, 5.55)	4.63 (3.65, 5.75)	0.004
Macrophage inflammatory protein-1α (µg/mL)	5.72 (4.57, 7.25)	5.07 (4.45, 6.25)	6.96 (5.95, 8.98)	0.139
Osteoprotegerin (ng/mL)	2.42 (0.35, 5.82)	0.26 (0, 3.45)	2.89 (1.91, 8.74)	< 0.001
RANKL (ng/mL)	58.41 (46.67, 80.56)	58.02 (47.5, 80.5)	69.39 (51.91, 88.91)	0.268
Tumor necrosis factor α (ng/mL)	3.5 (2.81, 4.17)	3.05 (2.49, 3.85)	3.82 (3.02, 5.18)	0.005
Glucose (mmol/L)	5.4 (5.1, 6)	5.3 (5, 5.55)	5.4 (5, 8)	< 0.001
Cholesterol (mmol/L)	5.15 (4.54, 5.67)	5.31 (5.03, 5.7)	4.7 (4.13, 5.28)	0.002
Triglyceride (mmol/L)	1.3 (0.9, 1.67)	1.21 (0.74, 1.68)	1.03 (0.84, 1.53)	0.447
High density lipoprotein cholesterol (mmol/L)	1.17 (0.98, 1.32)	1.24 (1.11, 1.46)	1.16 (0.97, 1.25)	0.001
Low density lipoprotein cholesterol^e^ (mmol/L)	3.18 (2.71, 3.82)	3.53 (3.05, 3.81)	3.01 (2.57, 3.66)	0.012
Osteopontin^f^ (µg/L)	4.4 (2.8, 12)	4.3 (2.55, 5.5)	6.7 (3.4, 37.9)	0.737
Resistin^e^ (ng/mL)	7.7 (5.9, 10.4)	7.3 (5.9, 12.8)	7.1 (5.6, 8.3)	0.096
Insulin-like growth factor-1^e^ (ng/mL)	126.7 (106.5, 156.3)	123.65 (103.2, 160.2)	113.4 (97.9, 141.3)	0.603
Leptin^e^ (µg/L)	4.9 (3.5, 8)	4 (3.2, 5.5)	7.9 (3.8, 14.8)	< 0.001
Adiponectin^e^ (ng/mL)	5385.8 (3917.8, 7394.8)	5541.05 (4198.4, 7344.7)	6377.7 (4729.4, 10,165.3)	0.406
CTX (mg/L)	0.31 (0.21, 0.41)	0.25 (0.21, 0.33)	0.28 (0.23, 0.38)	0.112
Osteocalcin (ng/mL)	15 (12, 18)	15 (12, 16)	15 (12, 19)	0.002
P1NP (µg/L)	31.4 (24.05, 40.55)	29.9 (24.05, 33.5)	30.9 (25.5, 43.5)	0.036
Insulin (µU/mL)	5.85 (4.55, 9.15)	5.3 (4.6, 8.25)	7 (4, 11.2)	0.004
C-reactive protein (mg/L)	1.15 (0.6, 2.9)	0.8 (0.5, 1.8)	1.7 (0.8, 3.6)	0.001

Fat variables	Mean (SD)			
Marrow fat at left femoral neck (mm^3^)	330.40 (482.50)	428.37 (611.16)	232.43 (276.73)	0.036
Visceral adipose tissue-L2/3 (mm^3^)	83,492.33 (39,572.74)	78,212.92 (36,599.33)	88,771.75 (41,980.31)	0.193
Subcutaneous adipose tissue-L2/3 (mm^3^)	60,684.92 (31,523.45)	49,717.35 (22,006.35)	71,652.48 (35,712.27)	< 0.001

*CTX* carboxy-terminal collagen crosslinks, *FN-BMD* bone mineral density at femoral neck, *LS-BMD* bone mineral density at lumbar spine, *P1NP* N-terminal propeptide of type 1 collagen, *RANKL* receptor activator of nuclear factor κ-B ligand

*Based on *t* test for values of mean (SD), Fisher’s test for *n* (%) and Mann–Whitney test for values of median (IQR)

^a^ *n* = 118

^b^ *n* = 117

^c^ *n* = 115

^d^ *n* = 50

^e^ *n* = 119

^f^ *n* = 101

### Relationships of Ethnicity with BMD

Indian men had significantly higher BMD at both sites compared to Chinese men (Table 2). Differences in BMD persisted between the two ethnic groups in the multivariate regression models. LS-BMD was 16.649 ± 4.974 mg/cm^3^ higher in Indian compared to Chinese men, while FN-BMD was 1.070 ± 0.029 times higher in Indian compared to Chinese men, after adjusting for potential confounders, including fat and bone biomarkers (Table 2).

**Table 2:** Effect of ethnicity on BMD outcomes

Outcomes		Non-adjusted model (Indian vs Chinese)		Adjusted model (Indian vs Chinese)	
			*p* values		*p* values
LS-BMD^a^	*β* (SE)	20.336 (4.749)	< 0.001	16.649 (4.974)	0.001
FN-BMD^b^	e^*β*^ (SE)	1.105 (0.032)	< 0.001	1.070 (0.029)	0.007

*FN-BMD* bone mineral density at femoral neck, *LS-BMD* bone mineral density at lumbar spine

^a^Model adjusted for smoking status, osteopontin, marrow fat at left femoral neck and visceral adipose tissue

^b^Model adjusted for smoking status, adiponectin and marrow fat at left femoral neck

### Predictors of LS-BMD

Smoking status and marrow fat at the left femoral neck showed significant bivariate association with LS-BMD (Supplementary Table 1). On multivariable analysis, marrow fat at the left femoral neck was found to be independently negatively associated with LS-BMD, with one standard deviation (SD) increase in marrow fat leading to a decrease in LS-BMD by 10.773 ± 2.928 mg/cm^3^ (*p* = < 0.001). VAT was also negatively associated with LS-BMD, each SD increase being associated with 5.055 ± 2.556 mg/cm^3^ decrease in BMD, though this did not reach statistical significance (*p* = 0.051). SAT was not significant in the model. While higher LS-BMD was observed in osteopontin quintile 2 (12.792 ± 7.368 mg/cm^3^, *p* = 0.086) compared to quintile 1, this did not reach statistical significance on multivariable analysis (Table 3). None of the other biomarkers were associated with LS-BMD.

**Table 3:** Predictors of BMD outcomes

Parameters of interest	LS-BMD		FN-BMD	
	*β* (SE)	*p* values	e^*β*^ (SE)	*p* values
Smoking status				0.287*
Never smoked	0.000	0.495*	1.000	
Ex-smoker	− 5.807 (5.648)	0.307	0.950 (0.030)	0.115
Smoker	5.597 (11.108)	0.616	0.995 (0.064)	0.936
Osteopontin				
Quintile 1	0.000	0.050*	–	–
Quintile 2	12.792 (7.368)	0.086	–	–
Quintile 3	6.974 (7.503)	0.355	–	–
Quintile 4	10.568 (7.341)	0.154	–	–
Quintile 5	− 7.550 (7.292)	0.303	–	–
Adiponectin				0.030*
Quintile 1	–	–	1.000	
Quintile 2	–	–	1.113 (0.044)	0.007
Quintile 3	–	–	1.117 (0.044)	0.006
Quintile 4	–	–	1.012 (0.041)	0.773
Quintile 5	–	–	1.072 (0.043)	0.084
Marrow fat at left femoral neck (standardised)	− 10.773 (2.928)	< 0.001	0.970 (0.014)	0.030
Visceral adipose tissue-L2/3 (standardised)	− 5.055 (2.556)	0.051	–	–
Indian* marrow fat at left femoral neck			0.909 (0.031)	0.007

*p value for Wald test of joint significance of regression coefficients

### Predictors of FN-BMD

In bivariate analysis, smoking status, marrow fat at left femoral neck, adiponectin and leptin showed significant associations with FN-BMD (Supplementary Table 1). Marrow fat at the left femoral neck remained negatively associated with FN-BMD in the multivariable analysis, with FN-BMD decreasing 0.954 ± 0.013 times (*p* = 0.001) with each SD increase in marrow fat. There was also significant interaction between ethnicity and marrow fat, with Indians having greater reduction in FN-BMD compared to Chinese for the same unit increase in marrow fat. Adiponectin was associated with FN-BMD, with significantly higher FN-BMD observed in adiponectin quintiles 2 (1.113 ± 0.044 times, *p* = 0.007) and 3 (1.117 ± 0.044 times, *p* = 0.007) compared to quintile 1. However, this increase was not observed with the highest levels of adiponectin (quintiles 4 and 5). Leptin was no longer significantly associated with FN-BMD after adjustment.

## Discussion

We found that ethnicity was an important predictor of LS-BMD and FN-BMD among older men, and the effect remained significant after adjusting for other variables of interest. Marrow fat at the left femoral neck and fat-associated biomarker adiponectin were found to be important predictors of BMD. Osteopontin and VAT were also associated with BMD with borderline significance.

Older Chinese had significantly lower volumetric BMD compared to older Indians in this study. This is consistent with our previous work among young men, where we demonstrated that Chinese had the lowest areal BMD [12]. These differences may be partly responsible for the high fragility fracture rates among Chinese in our population [11]. Similar ethnic variations in BMD in older men have been previously reported. The Osteoporotic Fractures in Men (MrOs) study [14] demonstrated ethnic variations in BMD among older US Black, White and Asian men, while a pooled analysis of several large cohorts across various populations similarly showed ethnic differences in BMD [10]. The ethnic differences in BMD observed were not explained by differences in body structure, fat, inflammation and bone metabolism. Hence, understanding the molecular mechanisms by which BMD is conserved at higher levels in some ethnic groups compared to others may be vital to identify possible targets for maintenance of bone health and treatment in osteoporosis.

Among the fat depots evaluated, marrow fat and VAT were associated with BMD. Marrow fat was negatively associated with BMD at both sites. This corresponds with findings from previous studies which demonstrated inverse relationships between bone marrow fat and BMD among various ethnic groups, including African American and Caucasian men and women [15], among Chinese women [26] and among Chinese men [27]. This increase in marrow fat could be a consequence of osteoporosis, with trabecular spaces created due to osteoporosis getting filled with fat. In addition, there is some evidence to suggest that marrow adipocytes and osteoblasts share a common precursor, and that these precursors preferentially differentiate into adipocytes instead of osteoblasts in osteoporosis [28, 29].

VAT was negatively associated with LS-BMD but not with FN-BMD. This is consistent with previous studies, which have reported similar inverse associations between VAT and BMD [16, 30]. However, the relationship between VAT and SAT with BMD has also been shown to be age, gender and bone-site specific [31]. These associations may be due to the metabolic effects of adipose tissue, through the expression of adipokines and inflammatory cytokines in patterns that could result in increased bone resorption [32, 33]. Compared to VAT, SAT is less metabolically active, which might partially explain the non-significant effect of SAT in our study [34].

We also evaluated a number of adipokines and inflammatory cytokines to elucidate their association with bone measures, and found only adiponectin to be significantly associated with FN-BMD. The positive relationship between adiponectin and BMD that we report is in contrast to findings from other studies which found negative associations of adiponectin with BMD and VAT [35–37]. Adiponectin is an adipose tissue-derived hormone that regulates glucose and fatty acid metabolism, and is negatively correlated with BMI [38, 39]. Since VAT is inversely associated with BMD, and adiponectin is inversely associated with VAT, a positive relationship between adiponectin and BMD is plausible. However, larger-scale longitudinal studies and animal experimental models will be needed to dissect the complex relationship between fat and bone.

Osteopontin was the bone marker associated with LS-BMD in our study, with borderline significance. Osteopontin has been shown to increase with increasing adiposity [40] and higher serum levels of osteopontin have been associated with lower BMD and higher risk of osteoporosis [41, 42]. Levels of several other biomarkers were significantly different between the ethnic groups, including vitamin D, osteoprotegerin, leptin, inflammatory markers (IL1α, IL6, CRP), insulin, glucose and cholesterol fractions. However, none of these markers showed significant associations with BMD at either site on bivariate and multivariable analysis. There was a positive association between HDL cholesterol and adiponectin, but no significant associations with other cholesterol fractions in our sample, which is similar to what has been previously reported [43, 44]. However, it is unlikely that this association has any implications on BMD in our study.

Limitations of our study warrant discussion. Our sample size was small, hence, we were unable to assess the complex mechanisms involved in bone metabolism and the interaction with serum biomarkers. However, this is the first Asian study involving older men from two major ethnic groups, representing around 37% of the world’s population, with detailed data on fat composition, bone and fat biomarkers in relation to BMD. There were differences in BMI between the two ethnic groups, and there may be errors in estimation of volumetric BMD due to fat. However, previous research suggests that fat has a smaller effect on BMD measured by QCT compared to DXA [45], and our findings are in line with previously reported ethnic differences in Singapore [12]. Other BTMs like bone-specific alkaline phosphatase were not measured, and data on dietary practices, which may influence body fat and bone mass, were not captured in our study, which are limitations. The cross-sectional nature of our study was another limitation. Larger prospective and intervention studies are needed to establish the effect of bone, fat and inflammatory markers on bone structure.

In conclusion, our study has shown that older Chinese men have significantly lower BMD at the femoral neck and lumbar spine as compared to Indian men. Other important predictors of BMD are marrow fat, VAT, adiponectin and osteopontin. Our findings highlight the increased risk of fragility fractures in older Chinese men, and warrant targeted ethnic-specific interventions to identify those at high risk to reduce fracture risk and manage osteoporosis. In addition, fat and fat-associated biomarkers were important independent predictors of lower BMD. This suggests that strategies aimed at the prevention and treatment of obesity and centralised fat deposition may be beneficial for improving bone health in the population, and not just cardiovascular risk.

## Electronic supplementary material

Below is the link to the electronic supplementary material.

Supplementary material 1 (DOCX 67 kb)

## References

[1] Odén A, McCloskey EV, Johansson H, Kanis JA (2013). Assessing the impact of osteoporosis on the burden of hip fractures. Calcif Tissue Int.

[2] International Osteoporosis Foundation (2014) The global burden of osteoporosis: a factsheet. Accessed date 2014

[3] IHME global burden of disease 2015. Institute of Health Metrics and Evaluation

[4] Johnell O, Kanis J, Oden A, Johansson H, De Laet C, Delmas P, Eisman J, Fujiwara S, Kroger H, Mellstrom D (2007). Predictive value of BMD for hip and other fractures (Journal of Bone and Mineral Research (2005) 20 (1185–1194)). J Bone Miner Res.

[5] Mithal A, Ebeling P, Kyer CS (2013) The Asia–Pacific regional audit. Epidemiology, costs and burden of osteoporosis in 2013. International Osteoporosis Foundation 10.4103/2230-8210.137485 PMC4138897 25143898

[6] Kanis JA, Oden A, McCloskey EV, Johansson H, Wahl DA, Cooper C (2012). A systematic review of hip fracture incidence and probability of fracture worldwide. Osteoporos Int.

[7] Cauley JA, Chalhoub D, Kassem AM, Fuleihan Gel H (2014). Geographic and ethnic disparities in osteoporotic fractures. Nat Rev Endocrinol.

[8] Cauley JA (2011). Defining ethnic and racial differences in osteoporosis and fragility fractures. Clin Orthop Relat Res.

[9] Looker AC, Melton LJ, Borrud LG, Shepherd JA (2012). Lumbar spine bone mineral density in US adults: demographic patterns and relationship with femur neck skeletal status. Osteoporos Int.

[10] Nam H-S, Shin M-H, Zmuda JM, Leung PC, Barrett-Connor E, Orwoll ES, Cauley JA (2010). Race/ethnic differences in bone mineral densities in older men. Osteoporos Int.

[11] Koh L, Saw S-M, Lee J, Leong K-H, Lee J (2001). Hip fracture incidence rates in Singapore 1991–1998. Osteoporos Int.

[12] Yang P, Lu Y, Khoo C, Leow M, Khoo E, Teo A, Lee Y, De Das S, Chong Y, Gluckman P (2013). Associations between ethnicity, body composition, and bone mineral density in a Southeast Asian population. J Clin Endocrinol Metab.

[13] Wang X-F, Duan Y, Beck TJ, Seeman E (2005). Varying contributions of growth and ageing to racial and sex differences in femoral neck structure and strength in old age. Bone.

[14] Marshall LM, Zmuda JM, Chan BK, Barrett-Connor E, Cauley JA, Ensrud KE, Lang TF, Orwoll ES (2008). Race and ethnic variation in proximal femur structure and BMD among older men. J Bone Miner Res.

[15] Shen W, Chen J, Gantz M, Punyanitya M, Heymsfield S, Gallagher D, Albu J, Engelson E, Kotler D, Pi-Sunyer X (2012). Ethnic and sex differences in bone marrow adipose tissue and bone mineral density relationship. Osteoporos Int.

[16] Choi HS, Kim KJ, Kim KM, Hur NW, Rhee Y, Han DS, Lee EJ, Lim S-K (2010). Relationship between visceral adiposity and bone mineral density in Korean adults. Calcif Tissue Int.

[17] Deurenberg-Yap M (2000) Body composition and diet of Chinese, Malays and Indians in Singapore: and their influence on cardiovascular risk factors. Landbouwuniversiteit Wageningen. Wageningen Agricultural University

[18] Nazare J-A, Smith JD, Borel A-L, Haffner SM, Balkau B, Ross R, Massien C, Alméras N, Després J-P (2012). Ethnic influences on the relations between abdominal subcutaneous and visceral adiposity, liver fat, and cardiometabolic risk profile: the International Study of Prediction of Intra-Abdominal Adiposity and Its Relationship With Cardiometabolic Risk/Intra-Abdominal Adiposity. Am J Clin Nutr.

[19] Reid IR (2002). Relationships among body mass, its components, and bone. BoneKEy-Osteovision.

[20] Biver E, Salliot C, Combescure C, Gossec L, Hardouin P, Legroux-Gerot I, Cortet B (2011). Influence of adipokines and ghrelin on bone mineral density and fracture risk: a systematic review and meta-analysis. J Clin Endocrinol Metab.

[21] Vasikaran S, Eastell R, Bruyère O, Foldes A, Garnero P, Griesmacher A, McClung M, Morris H, Silverman S, Trenti T (2011). Markers of bone turnover for the prediction of fracture risk and monitoring of osteoporosis treatment: a need for international reference standards. Osteoporos Int.

[22] Ding C, Parameswaran V, Udayan R, Burgess J, Jones G (2008). Circulating levels of inflammatory markers predict change in bone mineral density and resorption in older adults: a longitudinal study. J Clin Endocrinol Metab.

[23] Cauley JA, Barbour KE, Harrison SL, Cloonan YK, Danielson ME, Ensrud KE, Fink HA, Orwoll ES, Boudreau R (2016). Inflammatory markers and the risk of hip and vertebral fractures in men: the Osteoporotic Fractures in Men (MrOS). J Bone Miner Res.

[24] Shen W, Wang Z, Punyanita M, Lei J, Sinav A, Kral JG, Imielinska C, Ross R, Heymsfield SB (2003). Adipose tissue quantification by imaging methods: a proposed classification. Obes Res.

[25] Kvist H, Chowdhury B, Grangård U, Tylen U, Sjöström L (1988). Total and visceral adipose-tissue volumes derived from measurements with computed tomography in adult men and women: predictive equations. Am J Clin Nutr.

[26] Estrada K, Styrkarsdottir U, Evangelou E, Hsu Y-H, Duncan EL, Ntzani EE, Oei L, Albagha OM, Amin N, Kemp JP (2012). Genome-wide meta-analysis identifies 56 bone mineral density loci and reveals 14 loci associated with risk of fracture. Nat Genet.

[27] Travison T, Beck T, Esche G, Araujo A, McKinlay J (2008). Age trends in proximal femur geometry in men: variation by race and ethnicity. Osteoporos Int.

[28] Ahdjoudj S, Lasmoles F, Oyajobi BO, Lomri A, Delannoy P, Marie PJ (2001). Reciprocal control of osteoblast/chondroblast and osteoblast/adipocyte differentiation of multipotential clonal human marrow stromal F/STRO-1(+) cells. J Cell Biochem.

[29] Nuttall ME, Gimble JM (2000). Is there a therapeutic opportunity to either prevent or treat osteopenic disorders by inhibiting marrow adipogenesis?. Bone.

[30] Gilsanz V, Chalfant J, Mo AO, Lee DC, Dorey FJ, Mittelman SD (2009). Reciprocal relations of subcutaneous and visceral fat to bone structure and strength. J Clin Endocrinol Metab.

[31] Ng AC, Melton LJ, Atkinson EJ, Achenbach SJ, Holets MF, Peterson JM, Khosla S, Drake MT (2013). Relationship of adiposity to bone volumetric density and microstructure in men and women across the adult lifespan. Bone.

[32] Rosen CJ, Bouxsein ML (2006). Mechanisms of disease: is osteoporosis the obesity of bone?. Nat Clin Pract Rheumatol.

[33] Zhao LJ, Jiang H, Papasian CJ, Maulik D, Drees B, Hamilton J, Deng HW (2008). Correlation of obesity and osteoporosis: effect of fat mass on the determination of osteoporosis. J Bone Miner Res.

[34] Wajchenberg B, Giannella-Neto D, Da Silva M, Santos R (2001). Depot-specific hormonal characteristics of subcutaneous and visceral adipose tissue and their relation to the metabolic syndrome. Horm Metab Res.

[35] Lenchik L, Register T, Hsu F, Lohman K, Nicklas B, Freedman B, Langefeld C, Carr J, Bowden D (2003). Adiponectin as a novel determinant of bone mineral density and visceral fat. Bone.

[36] Michaëlsson K, Lind L, Frystyk J, Flyvbjerg A, Gedeborg R, Berne C, Zethelius B, Mallmin H, Söderberg S, Melhus H (2008). Serum adiponectin in elderly men does not correlate with fracture risk. J Clin Endocrinol Metab.

[37] Kontogianni MD, Dafni UG, Routsias JG, Skopouli FN (2004). Blood leptin and adiponectin as possible mediators of the relation between fat mass and BMD in perimenopausal women. J Bone Miner Res.

[38] Diez JJ, Iglesias P (2003). The role of the novel adipocyte-derived hormone adiponectin in human disease. Eur J Endocrinol.

[39] Ukkola O, Santaniemi M (2002). Adiponectin: a link between excess adiposity and associated comorbidities?. J Mol Med.

[40] Gómez-Ambrosi J, Catalán V, Ramírez B, Rodríguez A, Colina I, Silva C, Rotellar F, Mugueta C, Gil MJ, Cienfuegos JA, Salvador J, Frühbeck G (2007). Plasma osteopontin levels and expression in adipose tissue are increased in obesity. J Clin Endocrinol Metab.

[41] Chang I-C, Chiang T-I, Yeh K-T, Lee H, Cheng Y-W (2010). Increased serum osteopontin is a risk factor for osteoporosis in menopausal women. Osteoporos Int.

[42] Fodor D, Bondor C, Albu A, Simon S-p, Craciun A, Muntean L (2013). The value of osteopontin in the assessment of bone mineral density status in postmenopausal women. J Investig Med.

[43] Yamamoto Y, Hirose H, Saito I, Tomita M, Taniyama M, Matsubara K, Okazaki Y, Ishii T, Nishikai K, Saruta T (2002). Correlation of the adipocyte-derived protein adiponectin with insulin resistance index and serum high-density lipoprotein-cholesterol, independent of body mass index, in the Japanese population. Clin Sci (Lond Engl 1979).

[44] Park SH, Kim JY, Lee JH, Park HY (2010). Association between plasma adiponectin and high-density lipoprotein cholesterol in postmenopausal women. Clin Biochem.

[45] Yu EW, Thomas BJ, Brown JK, Finkelstein JS (2012). Simulated increases in body fat and errors in bone mineral density measurements by DXA and QCT. J Bone Miner Res.

