# Supplementary material for: Role of Fat and Bone Biomarkers in the Relationship Between Ethnicity and Bone Mineral Density in Older Men
Source: Calcif Tissue Int. 2017 Oct 20;102(1):64–72. doi: 10.1007/s00223-017-0342-8 (PMC5760609; doi:10.1007/s00223-017-0342-8)
Supplement: Supplementary file 1 — Supplementary material 1 (DOCX 67 kb) [file 223_2017_342_MOESM1_ESM.docx]

# Supplemental Table

**Supplemental Table 1. Bivariate associations with BMD outcomes**

| **Variables** | **LS-BMD** | | **FN-BMD** | |
| --- | --- | --- | --- | --- |
|  | **Coefficient (SE)** | **p-value** | **Coefficient (SE)** | **p-value** |
| **Race** |  |  |  |  |
| **Chinese** | **0.000** | **<0.001** | 1.000 | **<0.001** |
| **Indian** | **20.335 (4.749)** |  | **1.105 (0.032)** |  |
| Marital status |  |  |  |  |
| Never married | 0.000 | 0.510 | 1.000 | 0.674 |
| Currently married | -13.720 (14.135) |  | 0.946 (0.080) |  |
| Divorced/widowed | -16.113 (16.994) |  | 0.982 (0.100) |  |
| Education level |  |  |  |  |
| Up to primary | 0.000 | 0.200 | 1.000 | 0.400 |
| Secondary | 5.700 (7.316) |  | 0.965 (0.041) |  |
| Post-secondary | 5.781 (7.667) |  | 0.973 (0.044) |  |
| University | 2.723 (9.579) |  | 0.931 (0.053) |  |
| Refuse | 1.177 (15.230) |  | 1.106 (0.099) |  |
| Employment status |  |  |  |  |
| Working | 0.000 | 0.094 | 1.000 | 0.054 |
| Homemaker | -18.764 (27.481) |  | 0.921 (0.150) |  |
| Retired | -11.988 (5.153) |  | 0.956 (0.029) |  |
| Unemployed | -18.364 (12.737) |  | 0.820 (0.062) |  |
| Household income |  |  |  |  |
| Less than $2000 | 0.000 | 0.560 | 1.000 | 0.699 |
| $2000-$3999 | -2.327 (6.820) |  | 0.973 (0.041) |  |
| $4000-$5999 | 4.258 (7.835) |  | 0.943 (0.046) |  |
| $6000-$9999 | -8.613 (7.990) |  | 0.976 (0.048) |  |
| >$10000 | 9.875 (10.652) |  | 1.048 (0.073) |  |
| Refuse | 14.894 (19.865) |  | 0.909 (0.112) |  |
| Housing type |  |  |  |  |
| HDB 1-2 room | 0.000 | 0.793 | 1.000 | 0.403 |
| HDB 3 room | -2.924 (13.175) |  | 0.970 (0.075) |  |
| HDB 4 room | 2.239 (12.872) |  | 0.945 (0.072) |  |
| HDB 5 room | 3.627 (12.376) |  | 1.006 (0.073) |  |
| Private condo | 9.593 (13.637) |  | 1.057 (0.085) |  |
| Private house | -2.669 (12.757) |  | 0.963 (0.073) |  |
| Age (standardised) | -4.724 (2.567) | 0.068 | 1.002 (0.015) | 0.912 |
| BMI (Standardised) | 2.545 (2.540) | 0.318 | 1.013 (0.015) | 0.394 |
| Height (Standardised) | -1.148 (2.632) | 0.663 | 1.024 (0.015) | 0.118 |
| Alcohol consumption |  |  |  |  |
| No | 0.000 | 0.937 | 1.000 |  |
| Yes | -0.404 (5.116) |  | 0.975 (0.030) | 0.409 |
| Smoking status |  |  |  |  |
| Never smoked | 0.000 | 0.027 | 1.000 | 0.015 |
| Ex-smoker | -14.304 (6.213) |  | 0.900 (0.033) |  |
| Smoker | 15.108 (12.414) |  | 1.033 (0.076) |  |
| Family history of osteoporosis |  |  |  |  |
| No | 0.000 | 0.492 | 1.000 | 0.748 |
| Yes | 6.315 (9.155) |  | 0.983 (0.054) |  |
| Family history of fragility fracture |  |  |  |  |
| No | 0.000 | 0.373 | 1.000 | 0.200 |
| Yes | 6.649 (7.437) |  | 0.945 (0.042) |  |
| Skin score |  |  |  |  |
| 17-25 | 0.000 | 0.378 | 1.000 | 0.815 |
| 25-30 | -5.833 (5.795) |  | 0.989 (0.034) |  |
| >30 | 3.012 (6.701) |  | 0.974 (0.040) |  |
| Sun exposure score |  |  |  |  |
| Low | 0.000 | 0.436 | 1.000 | 0.088 |
| Moderate | 7.634 (6.833) |  | 1.039 (0.042) |  |
| High | 7.591 (9.358) |  | 1.124 (0.062) |  |
| Regular medication |  |  |  |  |
| No | 0.000 | 0.448 | 1.000 | 0.464 |
| Yes | 3.889 (5.109) |  | 0.978 (0.030) |  |
| Chest pain |  |  |  |  |
| No | 0.000 | 0.107 | 1.000 | 0.808 |
| Yes | 13.114 (8.069) |  | 1.012 (0.049) |  |
| CHD |  |  |  |  |
| No | 0.000 | 0.593 | 1.000 | 0.836 |
| Yes | -4.524 (8.443) |  | 0.989 (0.052) |  |
| Leg pain |  |  |  |  |
| No | 0.000 | 0.390 | 1.000 | 0.945 |
| Yes | 8.733 (10.130) |  | 0.996 (0.060) |  |
| Cerebrovascular disease |  |  |  |  |
| No | 0.000 | 0.402 | 1.000 | 0.946 |
| Yes | 10.638 (12.645) |  | 1.005 (0.076) |  |
| Hypertension |  |  |  |  |
| No | 0.000 | 0.765 | 1.000 | 0.333 |
| Yes | 1.630 (5.429) |  | 0.969 (0.032) |  |
| Diabetes mellitus |  |  |  |  |
| No | 0.000 | 0.648 | 1.000 | 0.389 |
| Yes | 3.144 (6.874) |  | 1.036 (0.043) |  |
| Dyslipidemia |  |  |  |  |
| No | 0.000 | 0.601 | 1.000 | 0.100 |
| Yes | 2.862 (5.455) |  | 1.057 (0.035) |  |
| History of fracture |  |  |  |  |
| No | 0.000 | 0.270 | 1.000 | 0.112 |
| Yes | -7.104 (6.416) |  | 0.942 (0.035) |  |
| Total activity level |  |  |  |  |
| Quintile 1 | 0.000 | 0.089 | 1.000 | 0.374 |
| Quintile 2 | 0.763 (7.920) |  | 1.010 (0.048) |  |
| Quintile 3 | -0.800 (8.004) |  | 1.013 (0.049) |  |
| Quintile 4 | 4.151 (7.920) |  | 0.993 (0.048) |  |
| Quintile 5 | 18.432 (7.920) |  | 1.083 (0.051) |  |
| Physical activity level - light |  |  |  |  |
| Quintile 1 | 0.000 | 0.670 | 1.000 | 0.340 |
| Quintile 2 | 7.133 (8.109) |  | 1.060 (0.051) |  |
| Quintile 3 | 6.237 (8.195) |  | 1.084 (0.052) |  |
| Quintile 4 | 9.743 (8.109) |  | 1.011 (0.048) |  |
| Quintile 5 | 12.654 (8.109) |  | 1.071 (0.051) |  |
| Physical activity level - moderate |  |  |  |  |
| Quintile 1 | 0.000 | 0.295 | 1.000 | 0.597 |
| Quintile 2 | -10.468 (7.720) |  | 0.982 (0.046) |  |
| Quintile 3 | -11.341 (8.450) |  | 0.923 (0.047) |  |
| Quintile 4 | 2.067 (7.944) |  | 0.984 (0.048) |  |
| Quintile 5 | -0.122 (7.944) |  | 0.969 (0.046) |  |
| Physical activity level - vigorous |  |  |  |  |
| Quintile 1 | 0.000 | 0.185 | 1.000 | 0.244 |
| Quintile 3 | 41.094 (27.609) |  | 1.111 (0.183) |  |
| Quintile 4 | 11.178 (6.496) |  | 1.068 (0.042) |  |
| Quintile 5 | 4.324 (6.496) |  | 0.977 (0.038) |  |
| Vitamin D |  |  |  |  |
| Quintile 1 | 0.000 | 0.353 | 1.000 | 0.707 |
| Quintile 2 | -1.354 (8.048) |  | 0.960 (0.047) |  |
| Quintile 3 | -11.930 (7.962) |  | 0.968 (0.047) |  |
| Quintile 4 | 2.724 (7.882) |  | 1.021 (0.048) |  |
| Quintile 5 | -7.325 (8.141) |  | 0.990 (0.048) |  |
| PTH |  |  |  |  |
| Quintile 1 | 0.000 | 0.218 | 1.000 | 0.738 |
| Quintile 2 | 3.259 |  | 1.035 (0.051) |  |
| Quintile 3 | -0.944 (7.835) |  | 1.010 (0.048) |  |
| Quintile 4 | -3.733 (7.755) |  | 0.986 (0.047) |  |
| Quintile 5 | 13.889 (7.922) |  | 1.046 (0.051) |  |
| PO4 |  |  |  |  |
| Quintile 1 | 0.000 | 0.221 | 1.000 | 0.872 |
| Quintile 2 | -12.398 (10.225) |  | 0.986 (0.066) |  |
| Quintile 3 | 7.451 (10.225) |  | 1.037 (0.069) |  |
| Quintile 4 | -9.310 (11.533) |  | 0.961 (0.072) |  |
| Quintile 5 | -12.607 (10.466) |  | 0.985 (0.069) |  |
| ALBgL |  |  |  |  |
| Quintile 1 | 0.000 | 0.793 | 1.000 | 0.605 |
| Quintile 2 | -11.007 (10.998) |  | 0.990 (0.067) |  |
| Quintile 3 | -5.632 (9.798) |  | 0.989 (0.061) |  |
| Quintile 4 | -12.268 (12.079) |  | 0.905 (0.067) |  |
| Quintile 5 | -2.299 (10.294) |  | 1.026 (0.065) |  |
| IFNgamma |  |  |  |  |
| Quintile 1 | 0.000 | 0.374 | 1.000 | 0.714 |
| Quintile 2 | -2.053 (7.967) |  | 1.010 (0.049) |  |
| Quintile 3 | 3.141 (7.967) |  | 0.991 (0.048) |  |
| Quintile 4 | 10.621 (8.054) |  | 1.057 (0.052) |  |
| Quintile 5 | 10.573 (8.054) |  | 1.019 (0.050) |  |
| IL1alpha |  |  |  |  |
| Quintile 1 | 0.000 | 0.141 | 1.000 | 0.676 |
| Quintile 2 | 10.801 (7.960) |  | 1.016 (0.050) |  |
| Quintile 3 | 4.916 (7.875) |  | 1.036 (0.051) |  |
| Quintile 4 | 7.838 (7.875) |  | 1.038 (0.050) |  |
| Quintile 5 | 20.071 (7.960) |  | 1.072 (0.052) |  |
| IL1beta |  |  |  |  |
| Quintile 1 | 0.000 | 0.414 | 1.000 | 0.721 |
| Quintile 2 | -8.309 (7.978) |  | 0.951 (0.046) |  |
| Quintile 3 | 5.373 (7.777) |  | 0.996 (0.047) |  |
| Quintile 4 | 0.095 (7.777) |  | 0.957 (0.045) |  |
| Quintile 5 | -7.668 (7.777) |  | 0.956 (0.045) |  |
| IL6 |  |  |  |  |
| Quintile 1 | 0.000 | 0.923 | 1.000 | 0.471 |
| Quintile 2 | -6.561 (8.172) |  | 0.938 (0.045) |  |
| Quintile 3 | -0.642 (8.085) |  | 0.979 (0.048) |  |
| Quintile 4 | -2.457 (8.004) |  | 0.965 (0.046) |  |
| Quintile 5 | -4.772 (8.267) |  | 0.923 (0.044) |  |
| MIP1alpha |  |  |  |  |
| Quintile 1 | 0.000 | 0.942 | 1.000 | 0.645 |
| Quintile 2 | -3.722 (8.090) |  | 0.959 (0.046) |  |
| Quintile 3 | 2.896 (8.090) |  | 1.020 (0.049) |  |
| Quintile 4 | 1.006 (8.272) |  | 0.964 (0.047) |  |
| Quintile 5 | -1.615 (8.090) |  | 0.965 (0.047) |  |
| OPG |  |  |  |  |
| Quintile 1 | 0.000 | 0.310 | 1.000 | 0.474 |
| Quintile 2 | 0.381 (8.035) |  | 1.024 (0.050) |  |
| Quintile 3 | 0.572 (8.035) |  | 0.986 (0.048) |  |
| Quintile 4 | -2.337 (8.120) |  | 1.005 (0.048) |  |
| Quintile 5 | 13.295 (8.035) |  | 1.071 (0.052) |  |
| RANKL |  |  |  |  |
| Quintile 1 | 0.000 | 0.388 | 1.000 | 0.983 |
| Quintile 2 | -8.275 (7.971) |  | 0.981 (0.047) |  |
| Quintile 3 | -5.445 (8.058) |  | 1.010 (0.049) |  |
| Quintile 4 | -11.272 (8.058) |  | 0.996 (0.049) |  |
| Quintile 5 | -15.215 (7.971) |  | 0.991 (0.048) |  |
| TNFalpha |  |  |  |  |
| Quintile 1 | 0.000 | 0.362 | 1.000 | 0.714 |
| Quintile 2 | -6.823 (7.810) |  | 1.017 (0.048) |  |
| Quintile 3 | -10.116 (8.244) |  | 1.021 (0.051) |  |
| Quintile 4 | -4.267 (7.964) |  | 0.973 (0.047) |  |
| Quintile 5 | 5.454 (8.050) |  | 1.040 (0.050) |  |
| Glucose |  |  |  |  |
| Quintile 1 | 0.000 | 0.273 | 1.000 | 0.336 |
| Quintile 2 | 12.818 (7.487) |  | 1.029 (0.046) |  |
| Quintile 3 | -1.107 (8.297) |  | 1.044 (0.053) |  |
| Quintile 4 | 11.841 (7.964) |  | 1.081 (0.051) |  |
| Quintile 5 | 5.792 (7.870) |  | 1.092 (0.052) |  |
| Cholesterol |  |  |  |  |
| Quintile 1 | 0.000 | 0.542 | 1.000 | 0.328 |
| Quintile 2 | -7.318 (8.008) |  | 0.931 (0.045) |  |
| Quintile 3 | -9.629 (8.094) |  | 0.925 (0.044) |  |
| Quintile 4 | -3.495 (8.008) |  | 0.995 (0.048) |  |
| Quintile 5 | -12.835 (8.094) |  | 0.972 (0.047) |  |
| TG |  |  |  |  |
| Quintile 1 | 0.000 | 0.425 | 1.000 | 0.867 |
| Quintile 2 | -10.611 (8.153) |  | 1.027 (0.050) |  |
| Quintile 3 | -14.659 (8.067) |  | 0.978 (0.048) |  |
| Quintile 4 | -4.485 (7.988) |  | 0.995 (0.047) |  |
| Quintile 5 | -7.021 (8.153) |  | 0.980 (0.048) |  |
| HDL |  |  |  |  |
| Quintile 1 | 0.000 | 0.497 | 1.000 | 0.655 |
| Quintile 2 | -2.868 (7.843) |  | 1.026 (0.049) |  |
| Quintile 3 | -9.846 (8.084) |  | 0.961 (0.047) |  |
| Quintile 4 | -4.223 (7.998) |  | 0.972 (0.047) |  |
| Quintile 5 | -13.175 (8.279) |  | 1.006 (0.050) |  |
| LDL |  |  |  |  |
| Quintile 1 | 0.000 | 0.768 | 1.000 | 0.070 |
| Quintile 2 | -9.914 (7.986) |  | 0.955 (0.044) |  |
| Quintile 3 | -7.085 (8.249) |  | 0.912 (0.043) |  |
| Quintile 4 | -5.064 (8.155) |  | 1.024 (0.048) |  |
| Quintile 5 | -8.407 (8.155) |  | 1.025 (0.048) |  |
| Cholesterol:HDL ratio |  |  |  |  |
| Quintile 1 | 0.000 | 0.153 | 1.000 | 0.631 |
| Quintile 2 | -2.630 (7.982) |  | 1.005 (0.048) |  |
| Quintile 3 | -12.748 (8.060) |  | 1.020 (0.049) |  |
| Quintile 4 | 7.767 (8.143) |  | 1.071 (0.053) |  |
| Quintile 5 | -3.285 (8.060) |  | 1.008 (0.049) |  |
| Osteopontin |  |  |  |  |
| Quintile 1 | 0.000 | 0.111 | 1.000 | 0.810 |
| Quintile 2 | 11.237 (8.354) |  | 0.985 (0.052) |  |
| Quintile 3 | 10.238 (8.466) |  | 0.977 (0.052) |  |
| Quintile 4 | 7.532 (8.466) |  | 0.954 (0.051) |  |
| Quintile 5 | -8.600 (8.354) |  | 0.943 (0.050) |  |
| Resistin |  |  |  |  |
| Quintile 1 | 0.000 | 0.143 | 1.000 | 0.425 |
| Quintile 2 | -10.358 (7.916) |  | 0.941 (0.045) |  |
| Quintile 3 | 0.367 (7.822) |  | 0.983 (0.045) |  |
| Quintile 4 | 2.974 (7.822) |  | 0.948 (0.044) |  |
| Quintile 5 | -14.164 (7.822) |  | 0.922 (0.044) |  |
| IFG1 |  |  |  |  |
| Quintile 1 | 0.000 | 0.816 | 1.000 | 0.849 |
| Quintile 2 | -4.151 (8.163) |  | 0.999 (0.048) |  |
| Quintile 3 | -0.773 (8.076) |  | 0.974 (0.046) |  |
| Quintile 4 | -8.873 (8.076) |  | 1.020 (0.049) |  |
| Quintile 5 | -1.863 (8.257) |  | 1.024 (0.051) |  |
| Leptin |  |  |  |  |
| Quintile 1 | 0.000 | 0.308 | 1.000 | 0.039 |
| Quintile 2 | 5.832 (8.139) |  | 0.931 (0.044) |  |
| Quintile 3 | -3.433 (7.960) |  | 0.887 (0.041) |  |
| Quintile 4 | 9.421 (7.960) |  | 0.981 (0.045) |  |
| Quintile 5 | 11.367 (8.046) |  | 1.003 (0.047) |  |
| Adiponectin |  |  |  |  |
| Quintile 1 | 0.000 | 0.534 | 1.000 | 0.013 |
| Quintile 2 | 9.358 (8.019) |  | 1.116 (0.051) |  |
| Quintile 3 | 13.899 (8.019) |  | 1.158 (0.052) |  |
| Quintile 4 | 7.792 (8.199) |  | 1.031 (0.047) |  |
| Quintile 5 | 8.981 (8.106) |  | 1.070 (0.050) |  |
| CTX |  |  |  |  |
| Quintile 1 | 0.000 | 0.658 | 1.000 | 0.179 |
| Quintile 2 | 1.769 (8.133) |  | 1.035 (0.049) |  |
| Quintile 3 | -2.780 (7.951) |  | 0.970 (0.046) |  |
| Quintile 4 | 8.829 (7.869) |  | 1.076 (0.050) |  |
| Quintile 5 | 0.094 (8.133) |  | 0.980 (0.047) |  |
| Osteocalcin |  |  |  |  |
| Quintile 1 | 0.000 | 0.529 | 1.000 | 0.928 |
| Quintile 2 | 9.002 (7.946) |  | 0.984 (0.047) |  |
| Quintile 3 | 5.997 (7.568) |  | 1.015 (0.046) |  |
| Quintile 4 | 6.571 (8.185) |  | 0.996 (0.049) |  |
| Quintile 5 | 13.250 (7.652) |  | 1.026 (0.048) |  |
| P1NP |  |  |  |  |
| Quintile 1 | 0.000 | 0.507 | 1.000 | 0.882 |
| Quintile 2 | 0.566 (8.000) |  | 1.044 (0.051) |  |
| Quintile 3 | -8.688 (8.000) |  | 1.035 (0.050) |  |
| Quintile 4 | -9.445 (8.180) |  | 1.039 (0.051) |  |
| Quintile 5 | 0.831 (8.000) |  | 1.048 (0.052) |  |
| Insulin |  |  |  |  |
| Quintile 1 | 0.000 | 0.426 | 1.000 | 0.392 |
| Quintile 2 | 8.624 (7.981) |  | 0.992 (0.047) |  |
| Quintile 3 | 9.413 (8.160) |  | 1.085 (0.053) |  |
| Quintile 4 | 15.165 (7.981) |  | 1.015 (0.048) |  |
| Quintile 5 | 11.168 (7.981) |  | 1.033 (0.050) |  |
| HOMAIR |  |  |  |  |
| Quintile 1 | 0.000 | 0.164 | 1.000 | 0.620 |
| Quintile 2 | 9.493 (8.066) |  | 1.000 (0.048) |  |
| Quintile 3 | 2.527 (7.889) |  | 1.065 (0.052) |  |
| Quintile 4 | 18.500 (7.889) |  | 1.042 (0.050) |  |
| Quintile 5 | 8.689 (7.889) |  | 1.039 (0.050) |  |
| hsCRP |  |  |  |  |
| Quintile 1 | 0.000 | 0.098 | 1.000 | 0.868 |
| Quintile 2 | 18.792 (7.942) |  | 1.002 (0.048) |  |
| Quintile 3 | 6.136 (7.764) |  | 0.986 (0.047) |  |
| Quintile 4 | 13.232 (7.850) |  | 0.979 (0.047) |  |
| Quintile 5 | 17.104 (7.764) |  | 1.031 (0.050) |  |
| SBP |  |  |  |  |
| Quintile 1 | 0.000 | 0.643 | 1.000 | 0.890 |
| Quintile 2 | -9.680 (7.948) |  | 0.971 (0.047) |  |
| Quintile 3 | -4.751 (8.115) |  | 0.965 (0.047) |  |
| Quintile 4 | -8.244 (8.209) |  | 0.960 (0.047) |  |
| Quintile 5 | -11.300 (8.028) |  | 0.995 (0.048) |  |
| DBP |  |  |  |  |
| Quintile 1 | 0.000 | 0.162 | 1.000 | 0.216 |
| Quintile 2 | -0.064 (7.973) |  | 0.903 (0.043) |  |
| Quintile 3 | 9.523 (7.734) |  | 0.978 (0.046) |  |
| Quintile 4 | 1.672 (8.164) |  | 0.930 (0.046) |  |
| Quintile 5 | -10.371 (7.887) |  | 0.944 (0.045) |  |
| Marrow fat at left femoral neck (standardized) | -13.955 (2.996) | <0.001 | 0.940 (0.013) | <0.001 |
| L23 VAT (standardized) | -4.290 (2.522) | 0.092 | 1.003 (0.015) | 0.824 |
| L23 SAT (standardized) | 0.441 (2.548) | 0.863 | 1.006 (0.016) | 0.698 |
